# Successful Percutaneous Coronary Stenting in End-Stage Liver Disease Patients Awaiting Liver Transplantation

**DOI:** 10.7759/cureus.76520

**Published:** 2024-12-28

**Authors:** Anoshia Raza, Philip Lim, Kajol Shah, Alexander Sudyn, Christine Gerula, Alfonso H Waller, Julius M Gardin, Marc Klapholz

**Affiliations:** 1 Cardiology, Newark Beth Israel Medical Center, Newark, USA; 2 Medicine, Rutgers University New Jersey Medical School, Newark, USA; 3 Medicine/Cardiology, Rutgers University New Jersey Medical School, Newark, USA; 4 Internal Medicine, Columbia University College of Physicians and Surgeons, New York, USA

**Keywords:** coronary artery angiography, coronary stents, liver transplant risk stratification, orthotropic liver transplant, primary percutaneous coronary intervention (pci)

## Abstract

Coronary artery disease (CAD) is associated with poor outcomes after orthotopic liver transplantation (OLT). We report on six high-risk end-stage liver disease (ESLD) patients who underwent percutaneous coronary intervention (PCI) with bare metal stents during the preoperative evaluation process. There was no mortality or major adverse cardiac event (MACE) within 90 days of OLT. These patients had advanced models for end-stage liver disease sodium (MELD-Na) scores (mean 24.5), thrombocytopenia (mean 70,500 µL⁻¹), and elevated international normalized ratio (INR; mean 2.0), who tolerated stent implantation followed by modified antiplatelet regimens. Percutaneous coronary intervention may facilitate listing with good OLT outcomes.

## Introduction

Significant coronary artery disease (CAD) left uncorrected is often a criterion for exclusion from orthotopic liver transplantation (OLT) due to poor outcomes associated with CAD [1-3]. While the diagnostic benefits of coronary angiography have been well-studied [4], data regarding transplantation outcomes after percutaneous coronary intervention (PCI) remain limited in the literature. Furthermore, patients with end-stage liver disease (ESLD) and advanced model for end-stage liver disease sodium (MELD-Na) scores in association with decreased platelet counts and elevated international normalized ratio (INR) are often not offered PCI because of the assumed prohibitive risk due to bleeding [4].

With the prevalence of cirrhosis increasing, CAD accounts for a major cause of mortality and morbidity in patients with ESLD despite rigorous preoperative cardiac testing [3-4]. This is likely due to shared traditional risk factors, including obesity, diabetes, and metabolic syndrome, and nontraditional risk factors, including inflammatory state, poor nutrition, and substance use [3]. Therefore, this highlights the importance of studying the outcomes of cirrhotic patients undergoing PCI. The objective of this study was to assess the outcomes of high-risk ESLD patients who underwent PCI. 

## Materials and methods

**Figure 1:**
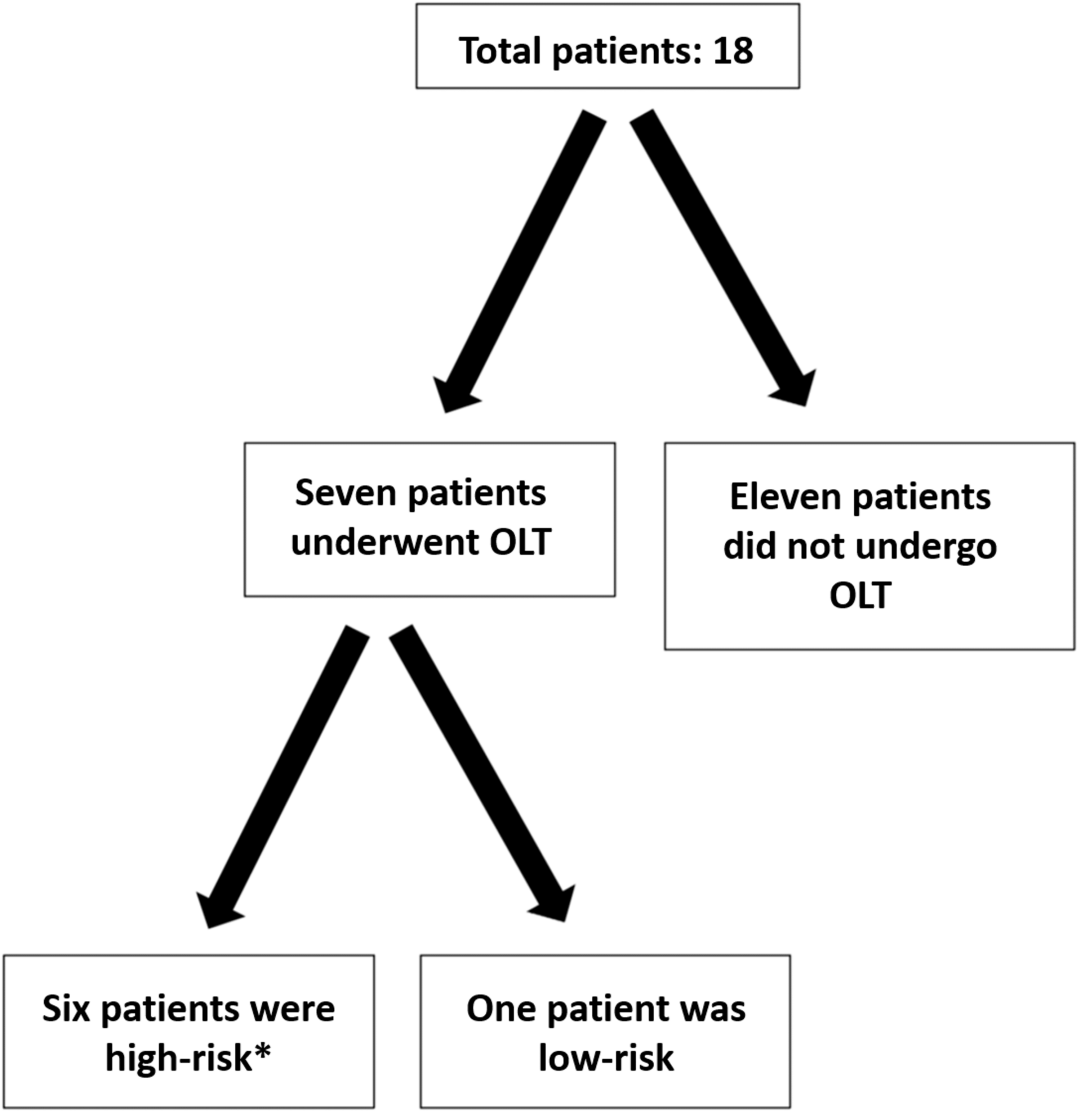
A flowchart outlining the study design We initially included 18 total patients who underwent PCI during the OLT evaluation process. Seven of those underwent successful OLT while 11 did not (due to various reasons listed in Table 1) and were excluded. Of the seven patients who underwent OLT, six were *high-risk patients, which was defined by MELD-Na scores above 20, platelet counts below 150,000 µL⁻¹, and INRs of at least 1.5. One of the seven patients was low-risk with a MELD-Na of only 12 and was excluded. OLT: orthotopic liver transplantation; PCI: percutaneous coronary intervention; MELD-Na: models for end-stage liver disease sodium; INR: international normalized ratio

After the approval from the Institutional Review Board of Rutgers University New Jersey Medical School, Newark, NJ (approval number: Pro2019000277), we conducted a retrospective chart review between August 2009 and August 2020. Patients at our institution were included if they underwent PCI during the OLT evaluation process (Figure 1). Patients were excluded if they did not undergo OLT or were not high-risk. A total of 18 patients were initially included; seven subsequently underwent successful OLT while 11 did not due to various reasons listed in Table 1. Of the seven patients who underwent OLT, we report on six high-risk patients who had MELD-Na scores above 20, platelet counts below 150,000 µL⁻¹, and INRs of at least 1.5, all of whom received bare metal stents (BMS). The seventh patient was low-risk with a MELD-Na score of only 12. We describe modified antiplatelet strategies tailored for each patient depending on the underlying severity of thrombocytopenia and hypocoagulability. Two independent reviewers were utilized to validate the data. 

**Table 1:** All patients who received PCI during OLT evaluation HTN: hypertension; MELD: model for end-stage liver disease; OLT: orthotopic liver transplantation; PCI: percutaneous coronary intervention; RAP: right atrial pressure

Patient	Received OLT	Reason for no OLT
Patient A	Yes	
Patient B	Yes	
Patient C	Yes	
Patient D	Yes	
Patient E	Yes	
Patient F	Yes	
Patient G	Yes	
Patient H	No	Currently listed and waiting
Patient I	No	No longer needed OLT due to low MELD score
Patient J	No	Died from septic shock 310 days months after PCI
Patient K	No	Currently listed and waiting
Patient L	No	Severe coagulopathy and septic shock; the patient refused treatment and requested comfort care. The patient expired nine days after PCI.
Patient M	No	Severe coagulopathy and septic shock. The family refused treatment and requested comfort care. The patient expired five days after PCI.
Patient N	No	No longer needed OLT due to low MELD score
Patient O	No	Died from septic shock 11 days after PCI
Patient P	No	Currently listed at an outside hospital
Patient Q	No	OLT was aborted due to a new diagnosis of very severe pulmonary HTN with RAP of 20 mm Hg. Of note, the patient was sent home to home hospice.
Patient R	No	No longer needed after hepatocellular carcinoma resected

## Results

All patients in this consecutive series tolerated PCI and underwent successful OLT without major adverse cardiac events (MACE) or mortality within 90 days of transplantation; MACE was defined as acute systolic heart failure, cardiac arrest, myocardial infarction (MI), and stroke. All six patients presented for OLT secondary to ESLD. Medical histories are detailed in Table 2.

**Table 2:** Coronary angiography and percutaneous coronary intervention prior to orthotopic liver transplantation BMS: bare metal stent; CAD: coronary artery disease; CKD: chronic kidney disease; D: diagonal; d-RCA: distal RCA; DAPT: dual antiplatelet therapy; DM: diabetes mellitus; EV: esophageal varices; GI: gastrointestinal; HBV: hepatitis B virus; HCC: hepatocellular carcinoma; HCV: hepatitis C virus; HE: hepatic encephalopathy; HLD: hyperlipidemia; HTN: hypertension; INR: international normalized ratio; LAD: left anterior descending; LCx: left circumflex; MACE: major adverse cardiac event; MELD-Na = modeled end-stage liver disease sodium; MI: myocardial infarction; NASH: non-alcoholic steatohepatitis; OLT: orthotopic liver transplantation; OM: obtuse marginal; p-LAD: proximal left anterior descending; PCI: percutaneous coronary intervention; RCA: right coronary artery; Femoral accesses were used earlier in the series and radial later in the series.

	Patient 1	Patient 2	Patient 3	Patient 4	Patient 5	Patient 6
Background	62-year-old White male patient	60-year-old Hispanic male patient	62-year-old Asian female patient	62-year-old White male patient	63-year-old Black male patient	55-year-old White male patient
Medical history	DM	HTN, DM	HTN, HLD, DM, prior CAD	Prior MI s/p BMS in m-LAD	HTN, HLD, DM, CKD, prior CAD	HTN, HLD
Social and family history	Smoker, family CAD	Smoker	Family CAD	Smoker	Smoker	None
Cirrhosis etiology	Cryptogenic	Alcohol, HCC	HBV	Alcohol	HCV	Alcohol
Pre-existing liver complications	Ascites, EV	Ascites, EV, HE	Ascites, EV, HE	Ascites, EV, HE	Ascites, EV	Ascites, EV, HE, GI bleed
Labs						
MELD-Na score	21	25	30	24	22	33
Hemoglobin (g/dL)	8.5	10.2	9.3	9.2	9.8	7.0
Platelets (x 10^3 ^µL^-1^)	76	120	61	144	65	30
INR	1.7	1.5	2.8	2.1	1.9	4.2
Serum creatinine (mg/dL)	1.0	1.2	0.7	0.8	1.4	1.0
Access*	Femoral	Femoral	Femoral	Femoral	Femoral	Radial
Stent location (stenosis %)	LCx (99%)	LCx (95%)	p-LAD (95%) Ostial RCA (95%)	D1 (95%)	p-LAD (79%) D1 (95%) RCA (69%)	p-LAD (80%)
Complications of PCI	Pseudoaneurysm	None	None	None	None	None
MACE within 90 days of PCI	None	None	None	None	None	None
MACE within 90 days of OLT	None	None	None	None	None	None
Mortality within 90 days of OLT	No	No	No	No	No	No

**Figure 2:**
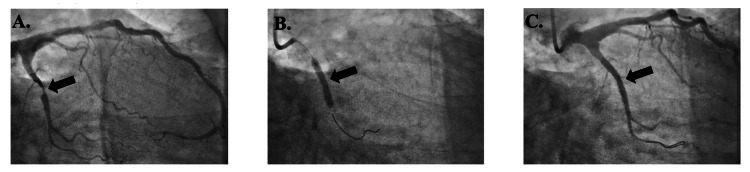
Patient 1's imaging reports Figure 2a. Lesion in LCx (arrow) before bare metal stent placement; Figure 2b. Lesion during bare metal stent placement (arrow); Figure 2c. Lesion after bare metal stent placement (arrow) Lcx: left circumflex artery

Patient 1 was a 62-year-old male with a MELD-Na score of 21, a platelet count of 76,000 µL⁻¹, and an INR of 1.7 who had a BMS placed in the left circumflex (LCx) coronary artery (Figure 2). The procedure was complicated by a pseudoaneurysm in the right femoral artery, which was addressed with an ultrasound-guided thrombin injection with no further complications. He was started on aspirin 81 mg daily and only one month of clopidogrel 75 mg daily. He underwent OLT 135 days after PCI and had no mortality or MACE.

**Figure 3:**
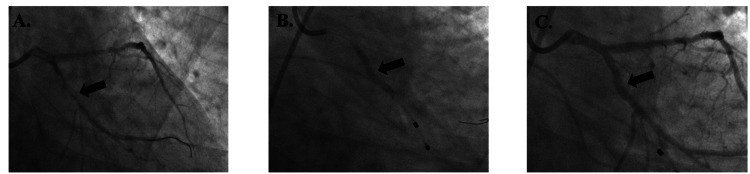
Patient 2's imaging reports Figure 3a. Lesion in LCx (arrow) before bare metal stent placement; Figure 3b. Lesion during bare metal stent placement (arrow); Figure 3c. Lesion after bare metal stent placement (arrow); Lcx: left circumflex artery

Patient 2 was a 60-year-old male with a MELD-Na score of 25, a platelet count of 120,000 µL⁻¹, and an INR of 1.5 who had a BMS placed in the LCx artery without complications (Figure 3). He was started on only prasugrel 10 mg daily for one month and then switched to aspirin 81 mg daily. He underwent OLT 57 days after PCI with no mortality or MACE.

**Figure 4:**
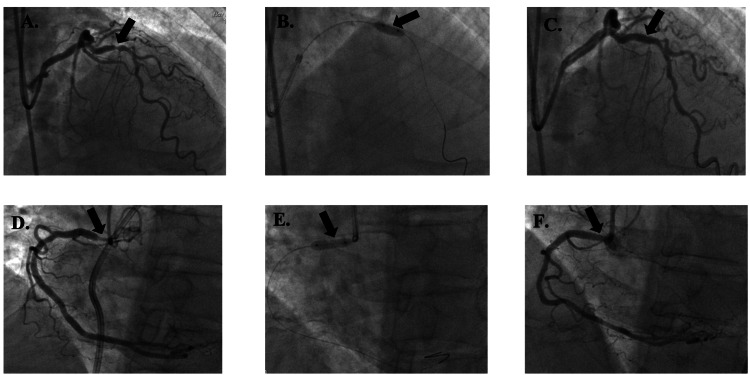
Patient 3's imaging reports Figure 4a. Lesion in p-LAD (arrow) before bare metal stent placement; Figure 4b. Lesion during bare metal stent placement in p-LAD (arrow); Figure 4c. Lesion after bare metal stent placement in p-LAD (arrow); Figure 4d. Lesion in ostial RCA (arrow) before bare metal stent placement; Figure 4e. Lesion during bare metal stent placement in ostial RCA (arrow); Figure 4f. Lesion after bare metal stent placement in ostial RCA (arrow) p-LAD: proximal left anterior descending artery; RCA: right coronary artery

Patient 3 was a 62-year-old female with a MELD-Na score of 30, a platelet count of 61,000 µL⁻¹, and an INR of 2.8 who had BMSs placed in the proximal left anterior descending (p-LAD) artery and ostial right coronary artery (RCA) without complications (Figure 4). She was started on aspirin 81 mg daily and received only one week of prasugrel 10 mg daily. She underwent OLT 54 days after PCI with no mortality or MACE. 

**Figure 5:**
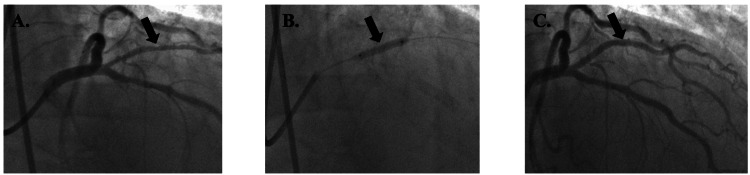
Patient 4's imaging reports Figure 5a. Lesion in D1 (arrow) before bare metal stent placement; Figure 5b. Lesion during bare metal stent placement (arrow); Figure 5c. Lesion after bare metal stent placement (arrow) D1: first diagonal branch

Patient 4 was a 62-year-old male with a MELD-Na score of 24, a platelet count of 144,000 µL⁻¹, and an INR of 2.1 who had a BMS placed in diagonal 1 (D1) without complications (Figure 5). He was started on aspirin 81 mg daily and only one month of clopidogrel 75 mg daily. He underwent OLT 312 days after PCI, which was complicated by septic shock. Three days after OLT, he had elevated troponins of 1.23, 1.14, and 0.82 but showed no clinical evidence of acute ischemia. He improved, with no mortality or MACE.

**Figure 6:**
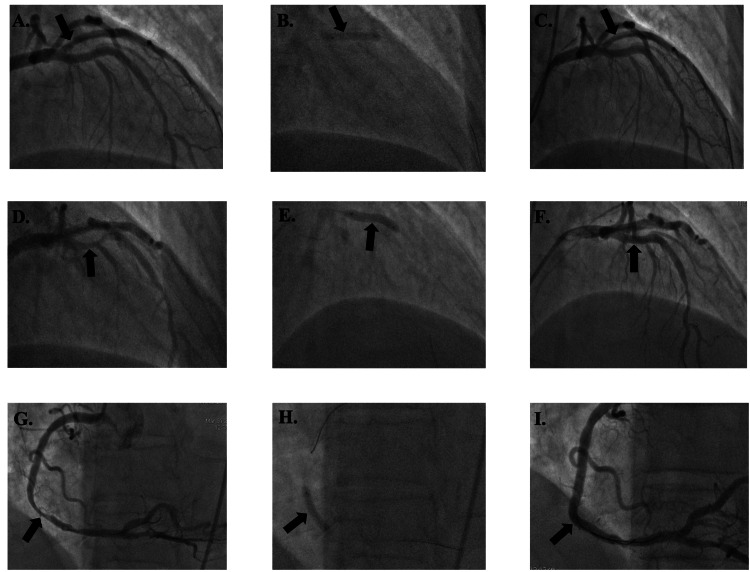
Patient 5's imaging reports Figure 6a. Lesion in D1 (arrow) before bare metal stent placement; Figure 6b. Lesion during bare metal stent placement in D1 (arrow); Figure 6c. Lesion after bare metal stent placement in D1 (arrow); Figure 6d. Lesion in p-LAD (arrow) before bare metal stent placement; Figure 6e. Lesion during bare metal stent placement in p-LAD (arrow); Figure 6f. Lesion after bare metal stent placement in p-LAD (arrow); Figure 6g. Lesion in RCA (arrow) before bare metal stent placement; Figure 6h. Lesion during bare metal stent placement in RCA (arrow); Figure 6i. Lesion after bare metal stent placement in RCA (arrow) D1: first diagonal artery; p-LAD: proximal left anterior descending artery; RCA: right coronary artery

Patient 5 was a 63-year-old male with a MELD-Na score of 22, a platelet count of 65,000 µL⁻¹, and an INR of 1.9 who had BMSs placed in the p-LAD, D1, and RCA without complications (Figure 6). He was started on aspirin 81 mg daily and only one month of clopidogrel 75 mg. He underwent OLT 542 days after PCI. One day after OLT, he was found to have elevated troponin levels of 0.67, 0.40, and 0.34. However, he had no clinical evidence of acute ischemia. He had no mortality or MACE. 

**Figure 7:**
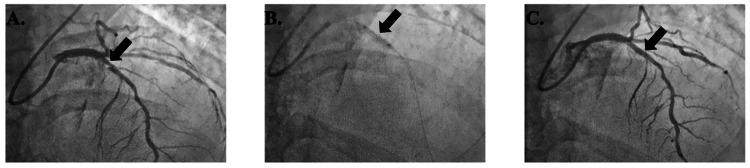
Patient 6's imaging reports Figure 7a. Lesion in p-LAD (arrow) before bare metal stent placement; Figure 7b. Lesion during bare metal stent placement (arrow); Figure 7c. Lesion after bare metal stent placement (arrow) p-LAD: proximal left anterior descending artery

Patient 6 was a 55-year-old male with a MELD-Na score of 33, a platelet count of 30,000 µL⁻¹, and an INR of 4.2 received a BMS in the p-LAD without complications (Figure 7). He was started only on aspirin 81 mg every other day until OLT, which he underwent 53 days after PCI with no mortality or MACE.

Medical therapy for all six patients is outlined in Table 3. 

**Table 3:** Medical therapy after PCI ASA: aspirin; OLT: orthotopic liver transplantation; PCI: percutaneous coronary intervention

Patient	Aspirin	Clopidogrel/Prasugrel therapy	Days Between PCI and OLT
Patient 1	ASA 81 mg daily	Clopidogrel 75 mg daily x 1 month	135
Patient 2	ASA 81 mg daily after 1 month	Prasugrel 10 mg daily x 1 month	57
Patient 3	ASA 81 mg daily	Prasugrel 10 mg daily x 1 week	54
Patient 4	ASA 81 mg daily	Clopidogrel 75 mg daily x 1 month	312
Patient 5	ASA 81 mg daily	Clopidogrel 75 mg daily x 1 month	542
Patient 6	ASA 81 mg every other day until OLT	None	53

## Discussion

We describe a selected group of patients who were appropriate candidates for OLT but were found to have significant coronary disease, which, if left untreated, would have excluded them from OLT. Seven out of the 18 patients who received PCI underwent successful OLT. Eleven did not undergo OLT; three no longer needed OLT due to low MELD scores, three were still on the list for OLT, one had OLT aborted due to severe pulmonary hypertension, and four did not make it to OLT due to mortalities. Of the mortalities, two died from septic shock, and two died from a combination of sepsis and bleeding complications. All mortalities occurred while patients were on palliative or hospice care.

Our six patients who underwent OLT had a median MELD-Na score of 24.5 (range 21-33), a platelet count of 70,500 µL⁻¹ (range 30,000-144,000), and an INR of 2.0 (range 1.5-4.2). After stent placement, these patients underwent successful OLT without mortality or MACE, highlighting the important utility of PCI prior to OLT.

Although data in the literature strongly associate the presence of CAD with poor OLT outcomes with a 50% post-transplant mortality and 80% morbidity among survivors [2], there is limited data comparing OLT outcomes in CAD patients who received PCI versus those who did not [5]. The few studies that have analyzed OLT outcomes after PCI report conflicting data [4-5]. Some studies show a decrease in all-cause OLT mortality after PCI, while other studies show the persistence of high risk for MACE-related OLT deaths after PCI [6-7].

Patients 4 and 5 had minimal elevation in their troponins within five days of OLT but without clinical evidence of ischemic electrocardiographic findings or any new wall motion abnormalities on echocardiography. These patients did not meet the criteria for MI based on the most recent universal definition [8]. It is worth noting that patients four and five in our series had the longest time periods between their PCI and OLT (542 and 312 days, respectively), which raises the question of appropriate follow-up in pre-OLT ESLD patients who have undergone PCI. Whether these patients should undergo repeat coronary angiography post PCI, if still within the pre-OLT period (perhaps at six months), deserves further study [9].

Decisions regarding dual antiplatelet therapy (DAPT) were individualized and based on overall clinical assessment to balance risks and benefits [10-11]. Whether utilization of clotting time, assessment of platelet aggregometry, or other strategies helps in clinical decision-making regarding DAPT post-PCI in ESLD patients requires further study [12]. Of note, guidelines suggest that shorter-duration DAPT can be considered for patients at lower ischemic risk with high bleeding risk [13-15].

## Conclusions

Our case series highlights the opportunity to offer PCI with BMS in patients with advanced liver disease, even in the presence of significant thrombocytopenia and elevated INR in order to facilitate OLT. While the risk of bleeding is high, the alternative for advanced ESLD patients is to be rejected for OLT with inevitable early mortality. Further research is needed to better define the safety profile of PCI and to develop specific recommendations for DAPT post PCI in both pre-OLT and post-OLT patients. Double-blinded studies with different antiplatelet regimens for post-PCI patients with cirrhosis are needed to better create a structured safety profile in these patients. Nonetheless, our case series suggests that careful clinical assessment and individualization of DAPT therapy can be effective in successfully managing these complex patients with multiple comorbidities to facilitate their eligibility for life-saving transplantation.

## References

[1] Diedrich DA, Findlay JY, Harrison BA, Rosen CB (2008). Influence of coronary artery disease on outcomes after liver transplantation. Transplant Proc.

[2] Plotkin JS, Scott VL, Pinna A, Dobsch BP, De Wolf AM, Kang Y (1996). Morbidity and mortality in patients with coronary artery disease undergoing orthotopic liver transplantation. Liver Transpl Surg.

[3] Reznicek E, Sasaki K, Montane B (2023). Outcomes of liver transplantation in patients with preexisting coronary artery disease. Transplantation.

[4] Khandait H, Jaiswal V, Hanif M (2023). Percutaneous coronary intervention outcomes in patients with liver cirrhosis: a systematic review and meta-analysis. J Cardiovasc Dev Dis.

[5] Patel SS, Lin FP, Rodriguez VA (2019). The relationship between coronary artery disease and cardiovascular events early after liver transplantation. Liver Int.

[6] Maddur H, Bourdillon PD, Liangpunsakul S (2014). Role of cardiac catheterization and percutaneous coronary intervention in the preoperative assessment and management of patients before orthotopic liver transplantation. Liver Transpl.

[7] Snipelisky DF, McRee C, Seeger K, Levy M, Shapiro BP (2015). Coronary interventions before liver transplantation might not avert postoperative cardiovascular events. Tex Heart Inst J.

[8] Thygesen K, Alpert JS, Jaffe AS, Chaitman BR, Bax JJ, Morrow DA, White HD (2018). Fourth universal definition of myocardial infarction (2018). J Am Coll Cardiol.

[9] Sharma V, Kleb C, Sheth C (2022). Cardiac considerations in liver transplantation. Cleve Clin J Med.

[10] Spence L, Russo M, Benbow J, Padilla L, Anderson W, Schrum L (2018). The risk of gastrointestinal bleeding is higher in patients with underlying cirrhosis on dual antiplatelet therapy. Am J Gastroenterol.

[11] Wu VC, Chen SW, Chou AH (2019). Dual antiplatelet therapy in patients with cirrhosis and acute myocardial infarction - a 13-year nationwide cohort study. PLoS One.

[12] Ostojic Z, Ostojic A, Bulum J, Mrzljak A (2021). Safety and efficacy of dual antiplatelet therapy after percutaneous coronary interventions in patients with end-stage liver disease. World J Cardiol.

[13] Levine GN, Bates ER, Bittl JA (2016). 2016 ACC/AHA guideline focused update on duration of dual antiplatelet therapy in patients with coronary artery disease: a report of the American College of Cardiology/American Heart Association Task Force on clinical practice guidelines: an update of the 2011 ACCF/AHA/SCAI guideline for percutaneous coronary intervention, 2011 ACCF/AHA guideline for coronary artery bypass graft surgery, 2012 ACC/AHA/ACP/AATS/PCNA/SCAI/STS guideline for the diagnosis and management of patients with stable ischemic heart disease, 2013 ACCF/AHA guideline for the management of ST-elevation myocardial infarction, 2014 AHA/ACC guideline for the management of patients with non-ST-elevation acute coronary syndromes, and 2014 ACC/AHA guideline on perioperative cardiovascular evaluation and management of patients undergoing noncardiac surgery. Circulation.

[14] Saracco M, Lavezzo B, Tandoi F (2023). Liver transplant outcome of cirrhotic patients treated with coronary stenting and early discontinuation of dual antiplatelet therapy. Dig Liver Dis.

[15] Remillard TC, Rockey DC (2021). From coronaries to cirrhosis: the role of percutaneous coronary intervention and dual antiplatelet therapy in end-stage liver disease. J Investig Med High Impact Case Rep.

